# “High or low Inferior Mesenteric Artery ligation in Laparoscopic low Anterior Resection: study protocol for a randomized controlled trial” (HIGHLOW trial)

**DOI:** 10.1186/s13063-014-0537-5

**Published:** 2015-01-27

**Authors:** Giulio Mari, Dario Maggioni, Andrea Costanzi, Angelo Miranda, Luca Rigamonti, Jacopo Crippa, Carmelo Magistro, Stefano Di Lernia, Antonello Forgione, Pietro Carnevali, Michele Nichelatti, Pierluigi Carzaniga, Francesco Valenti, Marco Rovagnati, Mattia Berselli, Eugenio Cocozza, Lorenzo Livraghi, Matteo Origi, Ildo Scandroglio, Francesco Roscio, Antonio De Luca, Giovanni Ferrari, Raffaele Pugliese

**Affiliations:** Dipartimento di Chirurgia Generale, AO Vimercate, Ospedale di Desio, Vimercate, Italy; Dipartimento di Chirurgia Generale e Videolaparoscopia, Ospedale Niguarda Ca’ Granda di Milano, Milan, Italy; Dipartimento di Chirurgia Generale, AO Provincia di Lecco, Ospedale di Merate, Lecco Italy; Dipartimento di Chirurgia Generale, Ospedale di Circolo di Varese, Varese, Italy; Dipartimento di Chirurgia Generale, AO Busto Arsizion, Ospedale di Tradate, Tradate, Italy

**Keywords:** Genito-urinary function, inferior mesenteric artery ligation;anastomotic leakage, rectal cancer, laparoscopic low anterior resection, oncological outcome

## Abstract

**Background:**

The position of arterial ligation during laparoscopic anterior rectal resection with total mesorectal excision can affect genito-urinary function, bowel function, oncological outcomes, and the incidence of anastomotic leakage. Ligation to the inferior mesenteric artery at the origin or preservation of the left colic artery are both widely performed in rectal surgery. The aim of this study is to compare the incidence of genito-urinary dysfunction, anastomotic leak and oncological outcomes in laparoscopic anterior rectal resection with total mesorectal excision with high or low ligation of the inferior mesenteric artery in a controlled randomized trial.

**Methods/design:**

The HIGHLOW study is a multicenter randomized controlled trial in which patients are randomly assigned to high or low inferior mesenteric artery ligation during laparoscopic anterior rectal resection with total mesorectal excision for rectal cancer. Inclusion criteria are middle or low rectal cancer (0 to 12 cm from the anal verge), an American Society of Anesthesiologists score of I, II, or III, and a body mass index lower than 30. The primary end-point measure is the incidence of post-operative genito-urinary dysfunction. The secondary end-point measure is the incidence of anastomotic leakage in the two groups. A total of 200 patients (100 per arm) will reliably have 84.45 power in estimating a 20% difference in the incidence of genito-urinary dysfunctions. With a group size of 100 patients per arm it is possible to find a significant difference (α = 0.05, β = 0.1555). Allowing for an estimated dropout rate of 5%, the required sample size is 212 patients.

**Discussion:**

The HIGHLOW trial is a randomized multicenter controlled trial that will provide evidence on the merits of the level of arterial ligation during laparoscopic anterior rectal resection with total mesorectal excision in terms of better preserved post-operative genito-urinary function.

**Trial registration:**

ClinicalTrials.gov Identifier: NCT02153801

Protocol Registration Receipt 29/5/2014.

**Electronic supplementary material:**

The online version of this article (doi:10.1186/s13063-014-0537-5) contains supplementary material, which is available to authorized users.

## Background

Colorectal cancer incidence and mortality rates vary around the world. Globally, colorectal cancer is the third most commonly diagnosed cancer in men and the second in women [1]. Surgical treatment of rectal cancer has changed radically in recent years. The introduction of total mesorectal excision [2], neo-adjuvant therapy protocols [3], and the laparoscopic approach [4] has made rectal cancer treatment a multidisciplinary management.

While laparoscopic resection of colon cancer has slowly gained acceptance worldwide, the role of laparoscopy in the treatment of rectal cancer is still controversial [4,5]. The laparoscopic treatment of rectal cancer raises specific issues related to its anatomical location: difficult exposure in a narrow pelvis, challenging nerve-sparing techniques, low intestinal transection, and total mesorectal excision [6,7]. Ligation of the inferior mesenteric artery at the origin and mobilization of the splenic flexure are not routinely done worldwide [8-11]. The need for well-designed studies to compare the different vascular approaches in rectal surgery has been clearly expressed [12-14].

The level of arterial ligation can affect genito-urinary function (injury to the superior hypogastric plexus), the extent (and yield) of lymphadenectomy, and distal colonic arterial perfusion (especially in older people, where distal colonic arterial perfusion could be deficient due to degenerative disease), and causesympathetic nerve injures [15-19]. Moreover, colonic stump blood supply and anastomosis tension are the main factors in developing leaks in rectal surgery [20-26] and are dependent on the level of ligation.

The aim of this study is to compare the incidence of genito-urinary dysfunction and, secondarily, the incidence of anastomotic leakage and the oncological outcomes in laparoscopic anterior rectal resection with total mesorectal excision with high or low ligation of the inferior mesenteric artery in a controlled randomized trial.

## Methods/design

### Study design

**Figure 1:**
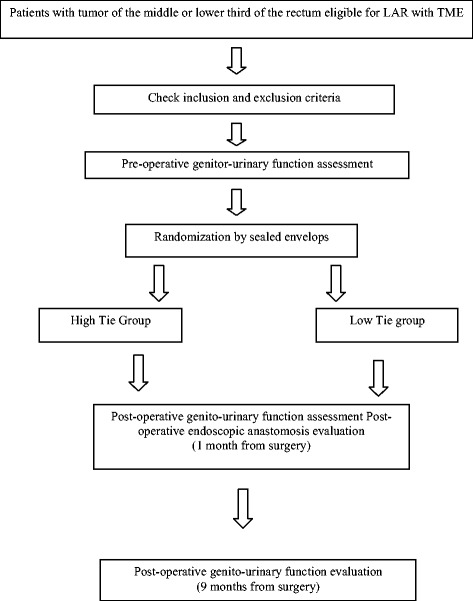
**Study flowchart.** LAR: Laparoscopic Anterior Resection. TME: Total Mesorectal Excision.

The HIGHLOW study is a multicenter controlled trial. Patients are randomly assigned to high or low inferior mesenteric artery during laparoscopy once the surgeon has confirmed intraoperatively, that all inclusion criteria are fulfilled and that the procedure is feasible. All participating surgeons have performed at least 20 laparoscopic procedures per year during the past 5 years. Randomization is performed using sealed envelopes (Figure 1).

### Primary and secondary endpoint measures

The primary end-point measure is the incidence of post-operative genito-urinary dysfunction compared with a preoperative base-line assessment in both groups. The secondary endpoint measures are the incidence of clinical or subclinical anastomotic leakage in the two groups and the oncological outcomes in both groups.

The incidence of clinical or subclinical anastomotic leaks will be determined either as the presence of a radiologically, endoscopically, or surgically proved fistula or as the presence of a subclinical leak observed by endoscopic examination 30 days after surgery in both groups.

Oncological outcomes will be assessed in terms of retrieved lymph nodes, number of positive lymph nodes on the root of the inferior mesenteric artery total number of lymph nodes of the mesorectum, and positive lymph nodes of the mesorectum, disease-free survival, overall survival, local recurrence, distant metastasis in both groups.

### Participating centers and

Five non-academic public Italian hospitals will enroll patients.

### Study population

The study population consists of patients with cancer of the lower two-thirds of the rectum, who are eligible for laparoscopic low rectal resection with total mesorectal excision.

Inclusion criteria are middle or low rectal cancer (from 0 to 12 cm from the dental line), an American Society of Anesthesiologists score of I, II, or III, and a body mass index less than 30. The study will include both preoperatively irradiated and non-irradiated patients.

Exclusion criteria are prior surgery on the abdominal aorta and proven arteriosclerosis of the inferior mesenteric artery and its branches.

The study will include both pre operatively irradiated and non-irradiated patients.

Exclusion criteria: prior surgery on the abdominal aorta, proven arterosclerosis of IMA and IMA branches.

### Ethical considerations

This study is conducted in accordance with the principles of the Declaration of Helsinki and “good clinical practice” guidelines. The study was approved by a Central Ethics Committee (list of names of the Ethical Body in Additional file 1). Prior to randomization written informed consent will be obtained from all patients.

### Preoperative evaluation

Patients with a preoperative diagnosis of adenocarcinoma of the middle or lower rectum and who satisfy the inclusion criteria will be asked to participate in the HIGHLOW trial. Once informed consent is obtained, investigations will include questionnaires for the International Index of Erectile Function [27], International Consultation on Incontinence Questionnaire [28], International Prostatic Symptoms Score, Female Sexual Function Inde [29] Uroflowmetry and ultrasound measurement of post-void bladder volume will be performed pre-operatively for all patients.

### Surgery

The following steps are required in all cases, independently of randomization.

#### Laparoscopic anterior rectal resection with total mesorectal excision (without pelvic cylindrical excixion)

The first step consist in the opening of the left part of the gastrocolic ligament and the division of the left part of transverse mesocolon. The splenocolic and phrenocolic attachments are then divided, achieving complete dissection of the left colonic angle. The pelvic peritoneum is opened below the sacral promontory and the hypogastric nerves are identified and preserved. The common iliac veins, the genitofemoral nerve, the gonadic vessels, and the left ureter are successively identified and preserved.

#### For high ligation

The opening of the peritoneum proceeds cephalad, towards the duodenojejunal angle of Treitz, and the mesenteric root is incised 1 cm below the inferior margin of the pancreas. The aortomesenteric window is opened wide and the inferior mesenteric vessels are exposed. The inferior mesenteric artery is ligated and divided at 2 cm from its origin. The inferior mesenteric vein is ligated and divided below the pancreatic margin

#### For low ligation

The opening of the peritoneum proceeds upward and then laterally towards the sigmoid colon. The leftcolic artery is identified and preserved while low ligation of the inferior mesenteric artery (the superior hemorrhoidal artery) is performed. Lymphadenectomy is performed medially along the inferior mesenteric artery as far as 2 cm from the aorta.

For both groups, dissection is then continued windowing Toldt’s and Gerota’s fascias up to the parietocolic gutter. Intra-pelvic dissection is carried out through standardized planes. Dissection of the rectum starts by incision of the peritoneal fold in the pelvis. Mesorectal excision starts posteriorly by dissection through Heald’s “holy plane”, it carries on towards the lateral region of the rectum, sparing the lateral part of the lateral rectal ligaments, and extends on the anterior side in front of Denonvilliers’ fascia.

### Post-operative evaluation

Colonoscopy will be performed 30 days after surgery to evaluate anastomosis (leakage, signs of ischemia) [26]. Accurate description and pictures of the anastomosis will be produced. Questionnaires for the Internation Index of Erectile Function [27], International Consultation on Incontinence Questionnaire [28], International Prostatic Symptoms Score, and Female Sexual Function Index [29] will be administrated, and uroflowmetry and ultrasound measurement of post-void bladder volume will be performed 1 and 9 months post-operatively. Retrieved lymph nodes will be collected from the histopathological examination, and the number of positive lymph nodes on the root of the inferior mesenteric artery, the total number of lymph nodes on the mesorectum, and the number of positive lymph nodes on the mesorectum will be recorded Oncological follow up will be carried out for 5 years, according to National Comprehensive Cancer Network Guidelines [30] Version 1.2015 Rectal Cancer/Surveillance (www.nccn.org).

### Sample size calculation

A two tail Fischer exact test applied to two cohorts of 100 patients each will have 84.45 power in estimating a 20% difference in the incidence of genitor-urinary dysfunctions. With a group size of 100 patients per arm it is possible to find a significant difference (α= 0.05, β= 0.1555).

With a drop-out estimated rate of 5%, the required sample size is 212 patients. If the number of drop-outs exceed 5%, we plan to ask the ethics committee to enroll more patients to be able to keep the power of analysis well above 80%.

### Statistical analysis

The Primary end-point measure will be evaluated using Fisher’s exact test (one-sided). The different incidences of genitor-urinary dysfunction according to sex and the results of each questionnaire will be evaluated using the Mann–Whitney *U test* and the *t-*test. Mc Nemar’s test will be used to evaluate changes in questionnaires results overtime. Statistical analysis will be performed in accordance with the intention-to-treat principle.

### Data collection and monitoring

Data will be collected daily using an Access database by one physician for each hospital and referred to a research fellow who will monitor the included data for all institutions. Patients will fill out questionnaires during pre and post-operative physical examinations. There will be regular contact between the study coordinators and the participating centers through scheduled meetings every 3 months. Uroflowmetry and ultrasound measurement of post-void bladder volume will be performed by the urologists of each institution, colonoscopies will be performed by endoscopists of each institution.

## Discussion

The discussion of the benefits or disadvantages between high tie and low tie in rectal surgery continues to be debated. Retrospective evaluations [9,31] have not produced a high level of significance regarding this issue. On the one hand, the risk of poor blood supply of the anastomosis could outweigh the oncological benefits of performing high ligation of the inferior mesenteric artery routinely, on the other hand a more powerful disease staging achieved with high ligation of the inferior mesenteric artery is found to be associated with an acceptable anastomotic leak rate. There is an increased risk of poor colonic stump blood supply when relying on the marginal artery alone, therefore, if this artery is not adequate, a more extended intestinal resection has to be performed, even if it is oncologically unnecessary. Performing lymphadenectomy extending to the origin of the inferior mesenteric artery even for low ligation provides data on the disease involvement of apical nodes. A significantly lower incidence of anastomotic leak in one or other group could indicate a technical policy for patients for which the vascular component might play a key role. Reduction of fistula in laparoscopic anterior rectal resection will produce a significant improvement in quality of life for these patients. Investigating pre and post-operatibegenitor-urinary function will provide a more complete assessment of the impact of laparoscopic anterior rectal resection on functional outcomes. Arterial ligation far from the hypogastric plexus could help in preserving pelvic autonomic functions, giving a better quality of life to patients.

We expect the low tie group to present a lower rate of genito-urinary function depression and post-operative fistula because of the better blood supply provided and better nerve sparing achieved.cpr

### Trial status

The study is not yet open for participant recruitment.

## Additional file

Additional file 1:
**Title and legend section.**

## Abbreviations

LAR: Laparoscopic Anterior Rectal Resection
TME: Total Mesorectal Excision
IMA: Inferior Mesenteric Artery
LCA: Left Colic Artery
ASA: American Society of Anesthesiologists
GU: Genito-Urinary
CRC: Colo-Rectal Cancer
BMI: Body mass Index
