# Supplementary material for: “High or low Inferior Mesenteric Artery ligation in Laparoscopic low Anterior Resection: study protocol for a randomized controlled trial” (HIGHLOW trial)
Source: Trials. 2015 Jan 27;16:21. doi: 10.1186/s13063-014-0537-5 (PMC4311448; doi:10.1186/s13063-014-0537-5)
Supplement: Additional file 1: — Title and legend section. [file 13063_2014_537_MOESM1_ESM.pdf]

Azienda Ospedaliera
Ospedale Niguarda Ca' Granda

COMITATO ETICO MILANO AREA C

A.O. OSPEDALE NIGUARDA CA' GRANDA
A.O. G. SALVINI GARBAGNATE MILANESE
A.O. OSPEDALE CIVILE DI LEGNANO
A.O. ISTITUTI CLINICI DI PERFEZIONAMENTO
ASL Milano 1

Responsabile del Procedimento: Prof. Bruno Cesana
Istruttore del Procedimento: Dr.ssa Mariangela Fumarola

Milano, 07 Aprile 2014

Piazza Ospedale Maggiore, 3, 20162
telefono: 02 6444.4689.4844
fax: 02 6444.4691
e-mail: comitatoeticoscientifico@ospedaleniguarda.it

Egregio Dottore
Raffaele Pugliese
Direttore S.C. di Chirurgia Generale
Oncologica e Mininvasiva
SEDE

Oggetto : **Richiesta di Parere HighLow**
Valutazione genito-urinaria in pazienti sottoposti a resezione anteriore di retto con escissione completa del mesoretto laparoscopica, con sezione della arteria mesenterica inferiore all' origine o distalmente all' emergenza della arteria colica sinistra
S.C. di Chirurgia Generale Oncologica e Mininvasiva
(Sperimentatore: Dr. Raffaele Pugliese)
Promotore: S.C. di Chirurgia Generale Oncologica e Mininvasiva dell'A.O. Ospedale Niguarda Ca' Granda

Si trasmette in allegato il parere del Comitato Etico-Scientifico di Milano Area C in merito allo studio in oggetto.

Questo documento non costituisce autorizzazione alla conduzione della sperimentazione presso l'A.O. Ospedale Niguarda Ca' Granda, per la quale è necessario attendere specifico Atto Amministrativo.

Distinti saluti

IL PRESIDENTE
Prof. Bruno Mario Cesana

Allegati

... NIGUARDA CA' GRANDA
Piazza Ospedale Maggiore, 3
20162 MILANO
COMITATO ETICO
MILANO AREA C

Sistema Sanitario Regione Lombardia

MARCHIO DEPOSITATO

TITOLO :
Valutazione genito-urinaria in pazienti sottoposti a resezione anteriore di retto con escissione completa del mesoretto laparoscopica, con sezione della arteria mesenterica inferiore all' origine o distalmente all' emergenza della arteria colica sinistra

SPONSOR o PROMOTORE:
S.C. di Chirurgia Generale Oncologica e Mininvasiva dell'A.O. Ospedale Niguarda Ca' Granda

SEDE:
S.C. di Chirurgia Generale Oncologica e Mininvasiva A.O. Ospedale Niguarda Ca' Granda - Dr. Raffaele Pugliese

VALUTATI I SEGUENTI DOCUMENTI:

- ✓ Elenco in lettera del Promotore del 13.01.2014 di cui copia in allegato quale parte integrante del presente Parere.

Il Comitato Etico ha espresso in data 28.03.2014 il seguente parere:

PARERE FAVOREVOLE CON RICHIESTA DI MODIFICHE ALLA CUI OTTEMPERANZA E' SUBORDINATO L'INIZIO DELLA SPERIMENTAZIONE

- Foglio Informativo: si chiede di completare il documento (pag. 3/5)
- Lettera al curante: modificare, specificando la differenza tra i due bracci di trattamento

Numero Registro **dei pareri : 158-032014**

Si rammenta anche che il Comitato Etico deve essere informato dell'inizio della sperimentazione e della sua conclusione o interruzione.

Il Responsabile della sperimentazione dovrà far pervenire una relazione annuale al Comitato Etico sullo stato della sperimentazione.

SI ALLEGA LISTA DEI COMPONENTI

[illegible] NIGUARDA CA' GRANDA
Piazza Ospedale Maggiore, 3
20162 MILANO
COMITATO ETICO
MILANO AREA C

MARCHIO DEPOSITATO

Chiarissimo Professore
Bruno Mario Cesana
Presidente Comitato Etico Milano Area C
A.O.Ospedale Niguarda Ca' Granda
Piazza Ospedale Maggiore, 3
20126 MILANO

Cpc
Alla Cortese Attenzione
Dott. Marco Trivelli
Commissario Straordinario
A.O.Ospedale Niguarda Ca' Granda
Piazza Ospedale Maggiore, 3
20126 MILANO

Milano, li 13 gennaio 2014

Oggetto: Richiesta di autorizzazione per la sperimentazione clinica dal titolo "Valutazione genito-urinaria in pazienti sottoposti a resezione anteriore del retto con escissione completa del mesoretto laparoscopica, con sezione della arteria mesenterica inferiore all'origine o distalmente all'emergenza della arteria colica sinistra"

Titolo dello studio:

Valutazione genito-urinaria in pazienti sottoposti a resezione anteriore del retto con escissione completa del mesoretto laparoscopica, con sezione della arteria mesenterica inferiore all'origine o distalmente all'emergenza della arteria colica sinistra.

Codice EUDRACT: 986

Con la presente il sottoscritto Raffaele Pugliese, Direttore SC di Chirurgia Generale Oncologica e Mininvasiva, desidera proporre a codesta Azienda Ospedaliera uno studio clinico, finalizzato al confronto tra due diversi livelli di legatura vascolare arteriosa, nel contesto dell'intervento di Resezione Anteriore di Retto Laparoscopica per tumore del retto extraperitoneale.

Lo studio clinico in oggetto verrà condotto presso la SC di Chirurgia Generale Oncologica e Mininvasiva dell'Azienda Ospedaliera Ospedale Niguarda Ca' Granda, diretta dal sottoscritto, Sperimentatore Principale, coadiuvato dai seguenti collaboratori: dott. G. Mari, Dott. M. Origi, doot. C. Magistero, dott. G. Ferrari, dott. S. Dilernia, dott. S. De Carli, dott. P. De Martini

Lo studio controllato,randomizzato, a gruppi paralleli multicentrico di cui in oggetto si svolgerà in 212 pazienti affetti da tumore del retto extraperitoneale, con indicazione ad intervento chirurgico di resezione anteriore del retto con escissione totale del tesoretto (*Totale Mesorectal Excision, TME*), afferenti alle seguenti 5 Strutture Complesse descritte:

AO VIMERCATE, PRESIDIO DI DESIO, CHIRURGIA GENERALE
AO OSPEDALE DI BUSTO ARSIZIO, PRESIDIO DI TRADATE, CHIRURGIA GENERALE
OSPEDALE DI CIRCOLO DI VARESE, DIPARTIMENTO DI CHIRURGIA GENERALE

A.O. NIGUARDA CA' GRANDA
Piazza Ospedale Maggiore, 3
20162 MILANO
COMITATO ETICO
MILANO AREA C

AO PROVINCIA DI ECCO, OSPEDALE DI MERATE, DIPARTIMENTO DI CHIRURGIA GENERALE
AO NIGUARDA CA GRANDA DIPARTIMENTO DI CHIRURGIA ONCOLOGICA E MINI-INVASIVA

Durata dello studio: 39 mesi
Durata dell' arruolamento: 30 mesi

nell'allegato CENTRI PARTECIPANTI.

Data di inizio studio: marzo 2014
Data di fine studio: luglio 2017

Una descrizione generale dello studio protocollo di studio è fornita nel PROTCOLLO in allegato presso il Comitato Etico.

L'obiettivo generale principale della sperimentazione è di confrontare la percentuale di insorgenza di disfunzioni genito-urinarie gravi in pazienti affetti da carcinoma del retto extraperitoneale, sottoposti a RAR + TME VL con legatura dell' Arteria Mesenterica Inferiore all' origine o distalmente all'emergenza dell' Arteria Colica Sinistra per valutare il migliore intervento chirurgico vascolare in termini di preservazione della funzionalità genito-urinaria.

L'endopoint primario è misurare l'incidenza di disfunzioni genito-urinarie gravi mediante incrocio dei risultati dei questionari di valutazione, del test uroflussodinamico e della valutzione ecografica del residuo post-minzionale, in pazienti sottoposti a RAR + TME VL con legatura dall'AMI distalmente all'origine dell' ACS (tecnica meno usata) rispetto alla RAR + TME VL con legatura dell'AMI all'origine (tecnica più frequente).

Gli *endpoints* secondari sono: vlutare e confrontare l'incidenza di fistole anastomotiche, associate ai due diversi tipi di approccio vascolare; valutare le potenziali differenti incidenze di disfunzioni genitali e/o urinarie, in dipendenza del sesso e delle risultanze dei singoli questionari, somministrati dallo sperimentatore.

Si allega alla presente domanda la documentazione sotto elencata:

- Protocollo di studio
- Sinossi del protocollo
- *Case Report Form* (CRF)
- Modulo di informazione e di consenso per il paziente
- Durata arruolamento 30
- Lettera al medico curante
- Lettera
- Elenco centri partecipanti
- CV Sperimentatore principale

Si dichiara che lo studio in oggetto verrà condotto secondo il Protocollo allegato.

Si accetta di condurre lo Studio in conformità alle G.C.P. e alle disposizioni normative vigenti.

Ci si impegna ad inviare al Comitato Etico ogni successivo emendamento al Protocollo.

Il proponente chiede di essere esonerato dal pagamento dei contributi economici richiesti per l'attività istruttoria del Comitato Etico, ai sensi del D.M. 17 dicembre 2004.

Dal punto di vista assicurativo gli obblighi di cui al Decreto Ministeriale 14 luglio 2009 non si applicano a questa tipologia di sperimentazione clinica a gruppi paralleli di confronto tra due tecniche chirurgiche correntemente utilizzate e validate.

A.O. NIGUARDA CA' GRANDA
Piazza Ospedale Maggiore, 3
20162 MILANO
COMITATO ETICO
MILANO AREA C

Infine, il proponente dichiara che non è previsto alcun compenso per lo sperimentatore principale per i soggetti arruolati.

Ringraziando per l'attenzione prestata a questa richiesta, inviamo distinti saluti.

Dott. R. Pugliese

A.O. NIGUARDA CA' GRANDA
Piazza Ospedale Maggiore, 3
20162 MILANO
COMITATO ETICO
MILANO AREA C

DICHIARAZIONE DEL RESPONSABILE DELLO STUDIO

Coordinatore/Responsabile dello studio osservazionale
(persona fisica che ha il compito di coordinare lo studio)

Pugliese Raffaele
(Nome e cognome)

Struttura pubblica o struttura no-profit nella quale opera il Coordinatore

Ospedale Niguarda ca' Granda di Milano

Azienda promotrice dello studio *(se diversa da quella già indicata)*

____
(specificare nome Azienda)

Io sottoscritto Dott. Raffaele Pugliese

in qualità di coordinatore dello studio osservazione dal titolo

Valutazione genito-urinaria in pazienti sottoposti a resezione anteriore del retto con escissione completa del mesoretto laparoscopica, con sezione della arteria mesenterica inferiore all'origine o distalmente all'emergenza della arteria colica sinistra.

DICHIARO che:

1. il/i farmaco/i è/sono prescritto/i nelle indicazioni d'uso autorizzate all'immissione in commercio (AIC) in Italia;
2. la prescrizione del farmaco/i in esame deve essere parte della normale pratica clinica;
3. la decisione di prescrivere il/i farmaco/i al singolo soggetto è del tutto indipendente da quella di includere il soggetto stesso nello studio (ove applicabile);
4. le procedure diagnostiche e valutative corrispondono alla pratica clinica corrente.

Milano, li 3/3/14

Firma del coordinatore ______________________

Firma del promotore *(ove applicabile)* ______________________

Si ricorda che per poter considerare uno studio di tipo osservazionale devono essere rispettate congiuntamente tutte le 4 condizioni sopra riportate

A.O. NIGUARDA CA' GRANDA
Piazza Ospedale Maggiore, 3
20162 MILANO
COMITATO ETICO
MILANO AREA C

Ai sensi della Determinazione AIFA 20 marzo 2008
"Linee guida per la classificazione e conduzione degli studi osservazionali sui farmaci"

SEDUTA DEL 28.03.2014 – PAGINA DELLE FIRME DEI COMPONENTI OBBLIGATORI

Cognome	*Nome*	*QUALIFICA*	*Struttura appartenenza (#)*	*FIRMA*
BARZAGHI	ADRIANA	RAPPRESENTANTE AREA PREFESSIONI SANITARIE	P	
BELLAVIA	GIUSEPPA	ESPERTO DISPOSITIVI MEDICI	A.O. NIGUARDA - MILANO	
BIGONI	ALESSANDRO	ESPERTO IN MATERIA GIURIDICA/MEDICO LEGALE	L.P.	
BREGNI	MARCO	CLINICO	A.O. BUSTO ARSIZIO	
BUSNACH	GHIL	CLINICO	L.P.	
CARNOVALI	MARINO	CLINICO	A.O. SALVINI - GARBAGNATE	
CELLA	SILVANO	FARMACOLOGO	UNIVERSITA' DEGLI STUDI DI MILANO	
CESANA	BRUNO	BIOSTATISTICO	UNIVERSITA' DEGLI STUDI DI BRESCIA	
CICCIO'	MIRELLA	ESPERTO IN MATERIA GIURIDICA/MEDICO LEGALE	L.P.	
COGLIATI	CLAUDIO	PEDIATRA	L.P.	
DE GASPERI	ANDREA	CLINICO	A.O. NIGUARDA - MILANO	
DE GIACOMO	COSTANTINO	PEDIATRA	A.O. NIGUARDA - MILANO	
ESPOSITO	ANNA	FARMACISTA SSR	A.O. NIGUARDA - MILANO	
GALLI	CARMEN	RAPPRESENTANTE VOLONTARIATO/ASSOCIAZIONISMO	L.P.	
GRASSI	ONORATO	ESPERTO IN BIOETICA	UNIVERSITÀ LUMSA DI ROMA	
LALATTA	FAUSTINA	ESPERTO GENETICA	IRCCS POLICLINICO MILANO	
LOLI	PAOLA	CLINICO	A.O. NIGUARDA - MILANO	
LORENZANO	ELETTRA	MEDICO MEDICINA GENERALE	L.P.	
MAGISTRELLI	VINCENZO	RAPPRESENTANTE AREA PREFESSIONI SANITARIE	P	
MARTINONI	ALESSANDRO	CLINICO	A.O. LEGNANO	
ONGARO	STEFANO	MEDICO MEDICINA GENERALE	L.P.	
ROFFI	LUIGI	CLINICO	A.O. I.C.P. - MILANO	
SCAGLIONE	FRANCESCO	FARMACOLOGO	UNIVERSITA' DEGLI STUDI DI MILANO	
SCANNI	ALBERTO	CLINICO	L.P.	
SCIASCERA	ALBA	CLINICO	A.O. LEGNANO	
TURAZZA	FABIO	CLINICO	A.O. NIGUARDA - MILANO	
VENTURELLI	GABRIELA	FARMACISTA SSR	A.O. NIGUARDA - MILANO	

(#) P = Collocato a riposo
L.P. = Libero Professionista

A.O. NIGUARDA CA' GRANDA
Piazza Ospedale Maggiore, 3
20162 MILANO
COMITATO ETICO
MILANO AREA C

MARCHIO DEPOSITATO

SEDUTA DEL 28.03.2014 – PAGINA DELLE FIRME

COMPONENTI IN RELAZIONE ALLA STRUTTURA

Cognome	Nome	QUALIFICA	STRUTTURA DI APPARTENENZA	FIRMA
DI MAGGIO	ANTONIO	DIRETTORE SANITARIO	ASL MILANO 1 E PROVINCIA	
MALNIS	DANIELA	SOSTITUTO DIR.SANITARIO	ASL MILANO 1 E PROVINCIA	
CATTANEO	SARAH	FARMACISTA	ASL MILANO 1 E PROVINCIA	
FOLCHINO	RACHELE	SOSTITUITO FARMACISTA	ASL MILANO 1 E PROVINCIA	
CALDARULO	TIZIANA	DIRETTORE SANITARIO	A.O. SALVINI - GARBAGNATE	
CARTONI	DAVIDE	SOSTITUTO DIR.SANITARIO	A.O. SALVINI - GARBAGNATE	
VIGANO'	MARIA TERESA	FARMACISTA	A.O. SALVINI - GARBAGNATE	
GOBBI	ADRIANA	SOSTITUITO FARMACISTA	A.O. SALVINI - GARBAGNATE	
LOMBARDO	MASSIMO	DIRETTORE SANITARIO	A.O. LEGNANO	
SCHIEPPATI	STEFANO	SOSTITUTO DIR.SANITARIO	A.O. LEGNANO	
DONDE'	PAOLA	FARMACISTA	A.O. LEGNANO	
VARALLI	LUCA	SOSTITUITO FARMACISTA	A.O. LEGNANO	
ODINOLFI	FULVIO EDOARDO	DIRETTORE SANITARIO	A.O. I.C.P. - MILANO	
DI MARINO	OSCAR	SOSTITUTO DIR.SANITARIO	A.O. I.C.P. - MILANO	
GIULIANI	GIANEMILIO	FARMACISTA	A.O. I.C.P. - MILANO	
MINA	SIMONA	SOSTITUITO FARMACISTA	A.O. I.C.P. - MILANO	
GENDUSO	GIUSEPPE	DIRETTORE SANITARIO	A.O. NIGUARDA - MILANO	
ELLI	GAETANO	SOSTITUTO DIR.SANITARIO	A.O. NIGUARDA - MILANO	
VENTURELLI	GABRIELA	FARMACISTA	A.O. NIGUARDA - MILANO	
ELLI	GAETANO	DIRETTORE SANITARIO	CENTRO CLINICO NEMO A.O. NIGUARDA	
VENTURELLI	GABRIELA	FARMACISTA	CENTRO CLINICO NEMO A.O. NIGUARDA	
PREGLIASCO	FABRIZIO	DIRETTORE SANITARIO	CASA DI CURA AMBROSIANA	
PAGANI	MARIA ANGELA	FARMACISTA	CASA DI CURA AMBROSIANA	
VIGNATI	EUGENIO CARLO MARIA	DIRETTORE SANITARIO	CLINICA SAN CARLO	
CARBONE	MARIANGELA	FARMACISTA	CLINICA SAN CARLO	

A.O. NIGUARDA CA' GRANDA
Piazza Ospedale Maggiore, 3
20162 MILANO
COMITATO ETICO
MILANO AREA C

MARCHIO DEPOSITATO

SEDUTA DEL 28.03.2014 – PAGINA DELLE FIRME

COMPONENTI IN RELAZIONE ALLA QUALIFICA

Cognome	*Nome*	*QUALIFICA*	*STRUTTURA DI APPARTENENZA*	*FIRMA*
CIBOLDI	MARCO	INGEGNERE CLINICO	A.O. NIGUARDA - MILANO	
FORGIONE	ANTONELLO	ESPERTO CLINICO TECNICHE DIGNOSTICHE	A.O. NIGUARDA - MILANO	
MANNA	GIULIA	ESPERTO IN NUTRIZIONE	A.O. NIGUARDA - MILANO	

A.O. NIGUARDA CA' GRANDA
Piazza Ospedale Maggiore, 3
20162 MILANO
COMITATO ETICO
MILANO AREA C
MARCHIO DEPOSITATO

Mariangela Fumarola

Da: Antonello Forgione **Inviato:** mar 4/8/2014 7:43
A: Mariangela Fumarola
Cc:
Oggetto: R: Parere in relazione alla qualifica
Allegati:

Vista la documentazione e gli atti esprimo parere favorevole e senza riserve allo svolgimento dello studio HIGH LOW TRIAL presentato dal Dr Pugliese.

Dr Antonello Forgione

Da: Mariangela Fumarola
Inviato: mar 01/04/2014 18.23
A: Antonello Forgione
Oggetto: Parere in relazione alla qualifica

Egr. dr. Forgione,
come anticipato telefonicamente, nella seduta del CE Milano Area C del 28.03.2014 era richiesta la sua presenza in relazione allo studio HIGH LOW TRIAL, presentato dal dr. Pugliese; ai fini del rilascio del parere, ci è necessario suo parere in qualità di esperto clinico di tecniche diagnostiche. Le invio in allegato lettera di trasmissione e sinossi del protocollo.
Cordiali saluti

dott.sa Mariangela Fumarola
Segreteria Comitato Etico Milano Area C
A.O. Ospedale Niguarda Ca' Granda
Piazza Ospedale Maggiore 3
20162 MILANO
Tel. 02-64444689
Fax. 02-64444691
email: *comitatoeticoscientifico@ospedaleniguarda.it;*
email: *mariangela.fumarola@ospedaleniguarda.it;*

A.O. NIGUARDA CA' GRANDA
Piazza Ospedale Maggiore, 3
20162 MILANO
COMITATO ETICO
MILANO AREA C
